# Dr. George Watt

**Published:** 1899-05

**Authors:** E. G. Betty

**Affiliations:** Cincinnati


### Dr. George Watt.

BY E. G. BETTY, D.D.S., CINCINNATI.

It was my good fortune, immediately upon graduating, to consummate an arrangement with Dr. Watt whereby I was to take charge of an operating chair in his office. I accordingly removed myself and my effects to the little town of Xenia, and was domiciled as a member of the doctor's household. I was a mere stripling and had scarcely ever been away from the fostering care of the paternal roof, and when I realized that home ties had been severed, the Rubicon crossed, and the great plain of my future stretched out before me, I was appalled, and had I been a girl I would undoubtedly have indulged in a good cry. As it was, my heart failed me and I told the doctor he would better get somebody else to do his operating for him, I did not feel equal to the occasion.

He was seated in his large, tilt-back chair, just such a one as you have often seen in the office of some down-town merchant, reading that good, old-fashioned paper, the Cincinnati *Gazette*. He looked up at the sound of my timorous voice, and for the first time I obtained a good view of as kind and merry a pair of blue eyes as ever twinkled over the golden bow of a pair of spectacles. He looked long and steadily at the trembling boy before him, summing up the situation, and I can hear the rustle of the paper to this day as he laid down the *Gazette* and said, "Let's take a walk." That walk was just what I needed, and he knew it. The morning was cold and crisp, the sun shone down from an intensely blue sky upon a light snow which had fallen during the previous night; the air, rich with ozone, filled my lungs with a draught as exhilarating as wine, and, ere half an hour had passed, a wonderful metamorphosis had taken place—the homesick lad was now an ambitious youth, ready to do or dare.

From that day forth a new life had begun for me, and under favorable conditions, too; for, though I did not then appreciate it, I have since learned to realize that I was associated with a man of broad attainment and correct habits of mind. He was brim-full of humor at nearly all times, and, no matter how dull the subject, how abstruse the point, he never failed to illustrate with a witty remark or humorous story which invariably relieved the tedium of hard work and prevented the mind from clogging. In all my acquaintance I can recall no one more tactful than Dr. Watt. He observed keenly, analyzed carefully, summed up with the skill of a practiced logician, and knew just the moment when "a word fitly spoken is like apples of gold."

Of Scotch ancestry, he was by nature argumentative, but not quarrelsome; talkative, without garrulity, and furthermore possessed the virtue of always having something to say.

He had a rare sense of justice and his sympathies were always with the under dog.

Nothing pleased him better than to spring a surprise upon you in the way of a new fact or to introduce an old friend with a new face and see what you thought of it.

Dr. Watt was not only a great reader, but he was a keen and consistent thinker. Nothing escaped his observation, for his eyes were quick to see and his well-trained mind classified and arranged its vast store of knowledge, while his discriminating judgment enabled him to use this ponderous power to the best advantage. Woe to an adversary in debate should he not be well fortified with an array of well-established fact.

He was the only man in the dental profession who could successfully handle and confute the late Dr. W. H. Atkinson, whose vagaries at one time occupied a large share of attention in the American Dental Association and other bodies, and much valuable space in the transactions and journals of that day.

Against these Dr. Watt now and then discharged a volley of satire and ridicule, so sharp, so scathing, so withering, that none but Atkinson could have held out so long. Dr. Atkinson's Angels and Volapuk, together with the animadversions of Benjamin Beryl Blynx, constitute a theme worthy of an Isaac D'Israeli, and deserves perpetuation as a curiosity of literature rather than a quarrel of authors. Atkinson's Volapuk contained within itself its own tubercle, though I am firmly convinced Dr. Watt's broadsides of grape and shrapnel but hastened what might otherwise have been a slow and painful death.

These attacks were in no wise acrimonious, but it was a sight worth miles of travel to look at Watt's face while Atkinson was going through his characteristic performance of injecting the grace of God with a syringe.

I hope some master hand will gather together the chronicles of this battle royal and give it to us in its entirety; it was a contest between giants, the like of which does not occur more than once in a generation.

Few writers in the ranks of the dental profession have wielded such a graceful pen; certainly none have excelled him in command of language. He was a close student of English literature and was familiar with all its classics; his remarkably retentive memory, enabling him to lay up vast stores of riches upon which he could draw at will. His diction reminded me much of Addison. Now and then of an evening by the cosy fireside he would give way to chatty reminiscence, his experience as a student, his trials as a medical practitioner in country districts, and through it all I could recognize a resemblance to dear old Sir Roger.

He once told me that his literary models were Goldsmith and Kirk White. Just think of it; Goldsmith and Kirk White.

What a contrast; what a gulf between them. Goldsmith, a Bohemian in every sense of the word, a poet who wrote for the world and for all time, who sounded the remotest depths of the human heart and set its strings to vibrating with eternal harmony.

Kirk White, a religious ascetic, long ago forgotten and whose name the most of you are now hearing for the first time.

I cannot account for the coupling together of these two men unless it be that Watt loved Goldsmith as the child of nature, while he revered Kirke White for his Calvinism.

Dr. Watt was gifted with that which few scientific men either possess, or if they have it, few cultivate—a lively imagination.

'Twas this which gave such rich coloring to his speech, an indescribable attraction in all he wrote. Many a gem has fallen from his lips, many a jewel adorned the editorial page. I have heard the complaint oft repeated, "This is not practical," or "that is not scientific," "we want facts." Poor, sodden fools, the pearl was there, but the swine saw it not.

Can any one recall throughout the whole length and breadth of dental literature anything to compare with "Lord Oxygen," and his worthy consort "Lady Hydrogen?" These are masterpieces of English composition, they bristle with scientific facts for the gradgrinds; the imagery is beautiful and delicate as the crystal flakes which fall from the winter sky.

I know of nothing with which to compare them unless it be Farraday's "Chemistry of a Candle," or Huxley's essay on "A Piece of Chalk."

Of the scientific nature of Geo. Watt's mind, his chemical theory of dental caries is ample evidence.

The careful construction and elaboration of that theory was the work of many years; for many years was it taught in the colleges and for near a generation was part and parcel of many a lecturer's stock in trade. And though in recent years the formulation of the germ theory through the labors of Mills and Underwood and Miller, in consonance with the germ theory of disease in general, has been of great benefit to dental science, it is possible the story has not all been told.

Farther and deeper investigation may yet explain the clinical features for which the chemical theory provided. There may or may not be found a bacillus to account for the presence of ammonia and its compounds in the mouth.

I have grave doubts that the microscope will do away with the science of organic chemistry. Until this is done Dr. Watt's chemical theory of dental caries, with all it implies, remains to be disproved. He may have builded wiser than we know.

In his private life Dr. Watt was eminently domestic; all his tastes gathered around the hearthstone. It was there the privil-edged friend became acquainted with the real inner character of the man. To have known him at such times is a memory to be cherished. The recollection of his good, kindly face will abide with me always.

I need scarcely say to those who knew him that he was a man of profound religious conviction, a condition of mind not at all inconsistent with an intellect trained in scientific induction. He firmly believed in the immortality of the soul. The heaven he longed for and looked forward to was one in which he should be permitted to peer into the infinite. As Sir John Lubbock words it: "The solution of problems which have puzzled us here; the acquisition of new ideas; the unrolling the history of the past; the world of animals and plants; the secrets of space; the wonders of the stars and of the regions beyond the stars. To become acquainted with all the beautiful and interesting spots of our own world would indeed be something to look forward to—and our world is but one of many millions. I sometimes wonder as I look away to the stars at night, whether it will ever be my privilege, as a disembodied spirit, to visit and explore them."

"I feel that to me," said Gregg, "God has promised not the heaven of the ascetic temper, or the dogmatic theologian, or of the subtle mystic, or of the stern martyr, ready alike to inflict and bear; but a heaven of purified and permanent affections—of a book of knowledge with eternal leaves, and unbounded capacity to read it—of those we love ever around us, never misconceiving us or being harassed by us—of glorious work to do and adequate facilities to do it—a world of solved problems, as well as of realized ideals."

It is meet that we do honor to the memory of such a man as Dr. Watt, and when we look upon this tablet I trust it will remind us of his lofty ideals and stimulate us to emulate a noble example.

